# Development and testing of a stifle function score in dogs

**DOI:** 10.3389/fvets.2022.895567

**Published:** 2022-07-25

**Authors:** Katie Gundersen, Darryl Millis, Xiaojuan Zhu

**Affiliations:** ^1^Department of Small Animal Clinical Sciences, College of Veterinary Medicine, University of Tennessee, Knoxville, TN, United States; ^2^Office of Information Technology, University of Tennessee, Knoxville, TN, United States

**Keywords:** stifle, function, score, dogs, rehabilitation

## Abstract

**Objective:**

The purpose of this study was to develop and test a quantitative stifle function score (SFS) in dogs with unilateral cranial cruciate ligament disease by combining clinical measures and functional tests. The objective of this study was to compare the proposed SFS to a symmetry index (SI) calculated from objective ground reaction forces (GRFs). We hypothesized that the SFS would have a strong correlation with SI.

**Methods:**

Dogs with surgically and nonsurgically treated unilateral cranial cruciate ligament rupture and dogs with no known musculoskeletal problems were included in the study. Each dog was scored using the SFS and trotted across a force platform to obtain GRFs and calculate the SI, based on vertical GRFs. Fourteen items were included in the SFS: limb use at a walk, limb use at a trot, lameness at a walk, lameness at a trot, stair climbing, sit-to-stand, dancing, pain response, stifle effusion, thigh circumference/muscle atrophy, stifle extension, stifle flexion, and cranial drawer/tibial thrust, with each item scored based on previously determined criteria. A perfect SFS would receive a score of 100.

**Results:**

Twenty-seven dogs were included in the study: twenty-one with unilateral cranial cruciate ligament disease and six control dogs. The mean SFS was 71.5 out of 100. To further characterize the association between SFS and SI the degree of gait asymmetry using SI was classified as <5%, 5.1–10%, 10.1–20%, 20.1–25%, and >25% difference between the pelvic limbs for all dogs. The mean SFS for each of the five categories were 97.8, 85.2, 65.4, 63, and 56.4, respectively. Correlation of SI and SFS was −0.863 (*p* < 0.0001). All of the individuals evaluated tests in the score were significantly correlated with SI except for pain response and stifle flexion. The SFS is in strong agreement with the SI, as confirmed by Bland–Altman analysis.

**Conclusion:**

The SFS had a significant correlation and agreement with the SI calculated from GRFs. This SFS may be a useful, simple, and inexpensive tool to use in a clinical environment to monitor progression during the rehabilitation and recovery process following unilateral cranial cruciate ligament rupture.

## Introduction

Cranial cruciate ligament rupture (CCLR) is commonly encountered in veterinary medicine. Rehabilitation is thought to be beneficial to dogs with CCLR (1–3). The therapy plan for each dog is determined by an initial evaluation of the dog's function and is altered as they are re-evaluated during their recovery to allow changes in their individual rehabilitation program. The main goals of physical rehabilitation are to return the dog to as normal function as possible and to be able to perform daily-life activities. Currently, monitoring a dog's progress during recovery is based primarily on subjective measures and alterations in rehabilitation are based on the dog's progress or by protocols with expected timeframes during recovery (4). In human medicine, there are several validated objective scoring systems to not only evaluate the knee after injury but also evaluate patient progression following reconstruction of the anterior cruciate ligament (5–7). Some of the items evaluated in these scoring systems include subjective evaluation of pain, swelling, giving way, walking, running, and ascending or descending stairs. Quantitative measures such as goniometry (8), thigh circumference (9, 10), static weight bearing (11), and force plate analysis (12) have previously been used to evaluate a patient's progress in veterinary medicine, but some of these tools are cost-prohibitive or not generally available in private practice. Force plate analysis is an objective measure of assessing lameness in dogs and is commonly used as an outcome measure after cruciate injury (13). However, this only provides objective information regarding weight-bearing at a walk or trot and not overall function. A validated stifle scoring system would be beneficial to evaluate patient progress for canine patients during recovery from CCLR. Two such systems have been proposed and are currently being evaluated (14–17). While both of these systems have been validated to differentiate dogs with and without cruciate ligament rupture, there has been a little comparison between these subjective scoring systems and objective measures, such as GRFs. The proposed scoring system in our study used items similar to validated scoring systems to evaluate knees in people, a variety of subjective and objective items that can be easily assessed in dogs, including some tasks that appear valuable based on other stifle scores in dogs, and was compared with GRFs.

The purpose of this study was to develop and test a quantitative stifle function score (SFS) in dogs with unilateral cranial cruciate ligament disease at different phases of disease by combining clinical measures and functional tests. The main objective of this study was to compare the SFS to a symmetry index (SI) calculated from measured GRFs from force plate analysis. We hypothesized that the SFS would have a strong correlation with the SI. Our overall goal is to provide a more comprehensive quantitative instrument using several items generally believed to be valuable in assessing the progress and outcomes of rehabilitation in dogs and to demonstrate that this SFS may be used to more closely assess the degree of weight-bearing as determined by GRFs.

## Materials and methods

### Dogs

Client-owned dogs were recruited and enrolled in the study with written owner consent. Inclusion criteria for the study were dogs weighing between 10 and 50 kg, ages 1–12, free of any major systemic illness as determined by physical examination, and appropriate blood tests and urinalysis if indicated. Two study populations were used: dogs with known unilateral cranial cruciate ligament disease were included regardless of when the injury occurred or whether or not surgery had been performed on the injured stifle (*n* = 21) and dogs with no evidence of any orthopedic disease served as controls (*n* = 6). Exclusion criteria included a body condition score >7/9, lameness in forelimbs or pelvic limbs unrelated to cranial cruciate ligament disease, bilateral cranial cruciate ligament disease, or neurologic abnormalities. This study was approved by the University of Tennessee Institutional Animal Care and Use Committee and was performed in accordance with AAALAC and USDA guidelines (No. 2765-0520).

### Kinetic analyses

A force platform (AMTI OR6-6, Watertown, MA, USA) was used to obtain GRFs which were then expressed as a percent of body weight. Four valid trials for each side of the dog were obtained at a trot. For a trial to be considered valid, dogs must have had no sudden deviation of gait, sudden head movements, turning of the head during gait, or any other motion that might affect the collection of kinetic data. Velocity and acceleration of the dog and handler were maintained between 1.7 and 2.1 m/s and ± 0.40 m/s^2^, respectively, using five photocells and a start-interrupt timer system. Mean peak vertical force values were used to identify weight-bearing asymmetry for each dog. SI was calculated using the equation: $SI=100\times \;abs\frac{\left(highest\;PVF-lowest\;PVF\right)}{\left(highest\;PVF+lowest\;PVF\right)}$ where a SI of 0% would represent perfect paired limb symmetry (18, 19). The degree of gait asymmetry using SI was further classified as <5%, 5.1–10%, 10.1–20%, 20.1–25%, and >25% difference between pelvic limbs.

### Stifle function score

Each dog was scored using the SFS (Supplementary Appendix 1) by the same blinded evaluator (DM). Fourteen individual tests were included in the score: limb use at a walk, limb use at a trot, lameness at a walk, lameness at a trot, stair climbing, sit-to-stand, dancing, pain response, stifle effusion, thigh circumference/muscle atrophy, stifle extension, stifle flexion, and cranial drawer or tibial thrust. The score ranged from 0 to 100, with a total score of 100 being perfect. The scoring details of each individual test are described in detail in Functional Tests and Clinical Measures. The entire SFS protocol can be viewed in Supplementary Appendix 1.

#### Functional tests

Limb use at a walk and trot were scored separately from 0 to 10: 10 = No lameness and weight-bearing on all strides, 6 = lame but weight-bearing on >95% of strides, 4 = lame but weight-bearing on >50 and <95% of strides, 2 = lame but weight-bearing on <50 and >5% of strides, and 0 = continuous non-weight-bearing lameness or weight-bearing on <5% of strides. Lameness at a walk and trot were scored separately from 0 to 10: 10 = normal locomotion, 8 = walks/trots with a slight (barely perceptible) lameness, but strides appear to have normal length, 6 = walks/trots with a mild lameness, but strides appear to have normal length, 4 = walks/trots with a moderate (obvious) lameness or a shortened stride length on the affected side when trotting, but is bearing weight on that limb, 2 = is intermittently non-weight-bearing on that limb when walking/trotting, and 0 = is completely non-weight-bearing on that limb when walking/trotting. The stance was scored from 0 to 10: 10 = stands with equal weight on both pelvic limbs, 6 = bears less weight on the affected pelvic limb or limb trembles when standing, 4 = puts limb down for balance but bears <10% of normal weight, and 0 = does not bear weight on an affected limb while standing. Stair climbing was scored from 0 to 5; 5 = no difficulty, 3 = slight difficulty climbing steps, 1 = skips steps or bunny hops, and 0 = cannot climb stairs. Sit-to-stand was scored from 0 to 5: 5 = easily goes from a sitting to a standing or a standing to sitting position/sits and rises squarely and symmetrically, 3 = sits or stands with some difficulty (slight hesitation or delay and mild asymmetry sitting or standing), 1 = sits or stands with difficulty (hesitation or delay and obvious asymmetry sitting or standing), and 0 = cannot sit or stand without assistance. Dancing was performed by lifting the dog's forelimbs off the ground, supporting them, and then moving them forward and backward. It was scored from 0 to 5: 5 = moves freely forward and backward, 3 = resists moving forward and backward, and 0 = unable to bear weight on pelvic limbs during forward and backward dancing motion.

#### Clinical measures

Pain response on palpation of the stifle joint capsule attachment sites were scored from 0 to 5: 5 = no pain response is elicited during palpation of the joint, 3 = mild pain response (i.e., head-turning) is elicited during palpation of the joint, 1 = moderate pain response (i.e., slight vocalization and increased reaction) is elicited during palpation of the joint, and 0 = severe pain response (i.e., immediate reaction, loud vocalization, and attempt to bite) is elicited during palpation of the joint. The amount of pressure placed on the joint capsule insertion sites was ~3 kg/cm^2^. To help assure reasonable clinical application of this amount of force, the evaluator practiced with a pressure threshold device until a consistent amount of pressure was obtained. Stifle effusion was scored from 0 to 5: 5 = no effusion of stifle, 3 = slight loss of patella ligament distinctness, 1 = patella ligament not distinct, and 0 = cannot distinguish patella ligament due to effusion. Thigh circumference/muscle atrophy was measured using a Gulick II tape measure and as previously described (9). It was scored 0–10: 10 = normal muscle mass, 6 = thigh girth is 1–5% smaller than the opposite limb, 4 = thigh girth is 6–10% smaller than the opposite limb, and 0 = thigh girth is >11% smaller than the opposite limb. Stifle extension and flexion were measured using a commercial goniometer and as previously described (8). Stifle extension was scored from 0 to 5: 5 = extension 160° or more, 3 = extension 150°-159°, 1 = extension 140°-149°, and 0 = extension <139°. Stifle flexion was scored from 0 to 5: 5= flexion 45° or less, 3 = flexion 46°-50°, 1 = flexion 51°-60°, and 0 = flexion >60°. Cranial drawer or tibial thrust was evaluated and scored from 0 to 5: 5 = <2 mm, 3 = 2–4 mm, 1 = 5–7 mm, and 0 = >7 mm. The direct cranial drawer was evaluated in dogs with no surgical correction or surgery with an extracapsular technique, while cranial tibial thrust was used to evaluate dogs following tibial plateau leveling osteotomy surgery or tibial tuberosity advancement surgery.

### Statistical analysis

The normality of data was assessed with a Shapiro–Wilk test and it was not normally distributed. Spearman's rank correlation, a nonparametric method, was used to measure the correlation coefficient between individual tests within the SFS and SI (IBM SPSS v.27). A Passing–Bablock regression was run to show the agreement between 1-SFS and SI. Bland–Altman analysis was then performed to also evaluate the agreement between 1-SFS and SI using SI as the gold standard (x-axis) and the difference between 1-SFS and SI as the y-axis (Medcalc v.20). *P* < 0.05 was considered significant.

## Results

**Figure 1:**
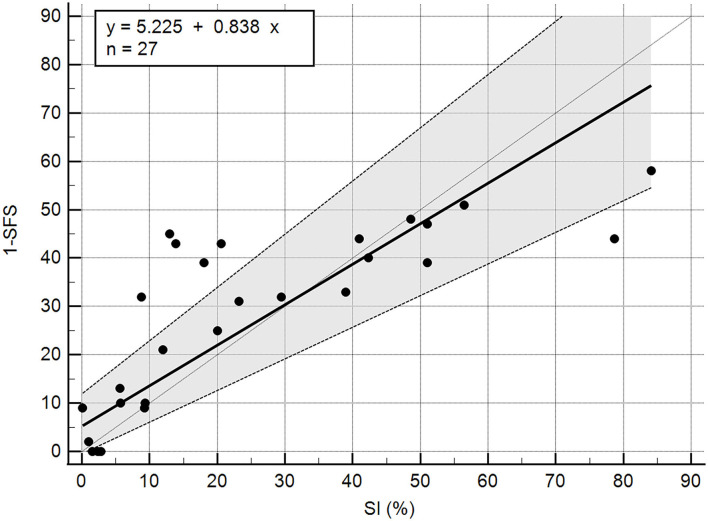
Passing Bablok regression of 1-SFS and SI (%).

A total of 27 dogs, 18 females and nine males, with a mean weight of 30.94 kg (median 29.5 kg; range 17.9–47 kg) were included in the analysis. The following breeds were represented: mixed breed (*n* = 14), Labrador Retriever (*n* = 5), German Shepherd (*n* = 3), Golden Retriever (*n* = 2), Standard Poodle (*n* = 1), Golden Doodle (*n* = 1), and Doberman (*n* = 1). Twenty-one dogs with unilateral cruciate ligament disease and six control dogs were included. Of the 21 affected dogs, 11 presented with acute unilateral complete cranial cruciate rupture (later confirmed during surgery), six had Tibial Plateau Leveling Osteotomy (TPLO) surgery performed 8 weeks prior, one had TPLO surgery 3 weeks prior, one had TPLO surgery 5 months prior, one had TPLO surgery 2 years prior, and one had suspected unilateral partial cranial cruciate tear. The mean SFS of this group was 71.56 out of 100. The mean SFS for each of the five categories of SI (<5%, 5–10%, 10–20%, 20–25%, and >25% difference) were 97.8, 85.2, 65.4, 63, and 56.4, respectively. Correlation of SI and SFS was −0.86 (*p* < 0.0001, Table 1). All of the individual tests in the SFS score were significantly correlated with SI except for pain response and stifle flexion (Table 1). The intercept of Passing–Bablok regression estimation was 5.225 with the 95% CI ranging from −0.4686 to 11.9159, and the slope estimation is 0.8377 with the 95% CI ranging from 0.6542 to 1.1006. The intercept 95% CI includes 0 and the slope 95% CI includes 1, indicating there is no significant difference between the intercept and 0 (the systematic difference between the two methods), and between the slope and 1 (the proportional differences between the two methods) (Figure 1).

**Table 1:** Spearman correlation between SI and other variables.

	**Spearman correlation with SI**	***P*-value (2-tailed)**
SFS	−0.863**	<0.0001
Limb use at walk (10)	−0.808**	<0.0001
Limb use at trot (10)	−0.804**	<0.0001
Lameness at walk (10)	−0.784**	<0.0001
Lameness at trot (10)	−0.819**	<0.0001
Stance (5)	−0.723**	<0.0001
Stairs (5)	−0.576**	0.002
Sit-to-stand (5)	−0.618**	0.001
Dancing (5)	−0.797**	<0.0001
Pain (5)	−0.353	0.071
Stifle effusion (5)	−0.422*	0.028
Muscle atrophy (10)	−0.568**	0.002
Stifle extension (5)	−0.590**	0.001
Stifle flexion (5)	0.05	0.803
Cranial drawer/tibial thrust (5)	−0.824**	<0.0001

^*^Correlation is significant at the 0.05 level (2-tailed).

^**^Correlation is significant at the 0.01 level (2-tailed).

**Figure 2:**
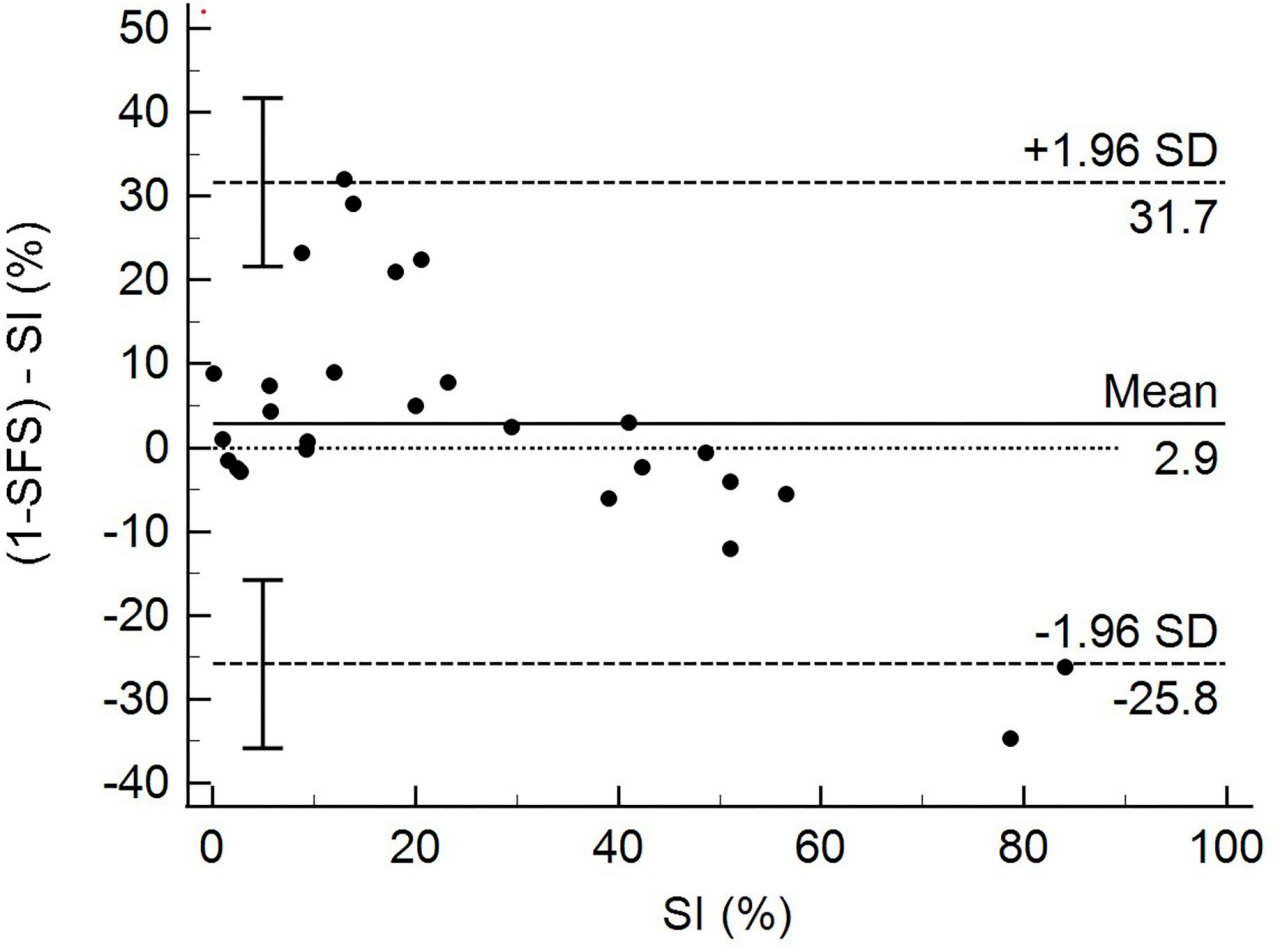
Graphic representation of Bland-Altman plot between (1-SFS) - SI (%) and SI %.

The total SFS was in strong agreement with SI, as confirmed by the Bland-Altman analysis (Figure 2). The 1-SFS vs. SI bias mean was 2.9 with an SD of 14.66. The 95% limit of agreement ranged from −25.802 to 31.67. The Bland–Altman plot showed that the SFS overestimated SI when the SI value range was 0–20 (more symmetric) and underestimated SI when SI > 40 (more asymmetric) (Figure 2).

## Discussion

The main purpose of this study was to develop and test a quantitative SFS in dogs with unilateral cranial cruciate ligament disease based on commonly used clinical parameters and functional tests and to compare this SFS with objective force platform analysis. Development of the score consisted of combining both clinical measures and functional tests to better assess a dog's overall stifle function. These items were chosen based on previous studies that evaluated stifle function following cruciate injury and clinical experience in evaluating the function of patients with cranial cruciate ligament disease (20). We accept our hypothesis that the SFS would have a strong correlation with SI based on objective GRFs. Correlation of SI and SFS was −0.86 (*p* < 0.0001).

Measurement of GRFs using a force platform was used to evaluate the SFS and is a potential weakness of this study. GRFs are a highly sensitive method of evaluating lameness, but may not be a true measure of functionality in patients with cruciate ligament disease. Unfortunately in veterinary medicine, there is no current gold standard for evaluation of a dog's overall function in regards to stifle disease. Knee function in people is commonly assessed using subjective criteria, and these scoring systems are in common use, but require the input of the patient in scoring many of the items. We chose to use GRFs to compare our SFS because it is an objective measure of weight-bearing, does not require patient input, and has excellent sensitivity and specificity in detecting lameness. The sensitivity of subjective or visual lameness scores is relatively low unless severe lameness is present (20).

To further assess the SFS, we tested the agreement between SFS and SI using Bland–Altman analysis, using SI as the gold standard test. This analysis showed good overall agreement between SFS and SI. The Bland–Altman analysis also showed that the SFS tended to be higher (overestimated function of the patient) when the SI value range was 0–20 (more symmetric) and tended to be lower (underestimated function of the patient) when SI > 40. This overestimation and underestimation may be due to limitations in the degree of function and distribution of the study population regarding the degree of disability of patients with CCLR. Some of the study dogs presented with acute CCLR that had not yet been surgically corrected or control patients. This resulted in very low SFS and higher SI for dogs with acute CCLR, and very high SFS and lower SI in normal dogs. More patients at various stages of recovery after surgery or further along in recovery with conservative management may have resulted in greater data spread with fewer extremes. Nevertheless, the SFS was able to discern dogs that were doing well in terms of function from those that still had significant mobility issues as a result of their CCLR. Another explanation for overestimating or underestimating the function of patients may be due to the weighing of various test items. For example, patients with poor limb use (percentage of strides that the patient bears some weight on the limb) also received low scores regarding lameness. While the intent of the SFS was to capture those patients with consistent use of the limb, yet still having various degrees of lameness, it also severely penalizes those dogs that have intermittent limb use as also being severely lame. Using GRFs as the comparison to our SFS could also explain the overestimation and underestimation seen in the analysis because the agreement of subjective lameness scores is greatest at either end of the lameness spectrum (i.e., no lameness or severe lameness) (20).

Individualized tests within the SFS were also evaluated to better assess items in the score to determine if each item contributes to the total functional score. Development of the score and deciding which items to include was based on previous studies that ranked evaluation methods for the canine stifle (21). The evaluation methods in that ranking included thigh circumference, sitting position, static weight-bearing, stifle range of motion, stair climbing, and visual evaluation of lameness (21). Based on our clinical experience, these evaluation methods and additional components were added to the SFS. In the proposed SFS, all the individual tests were significantly correlated with SI except for pain response and stifle flexion. The pain response test was based on palpation of the joint around joint capsule insertion sites rather than if the dog was painful throughout the range of motion or during hyperextension of the stifle. It is possible that many dogs were not painful on passive palpation of the stifle joint but might be with stifle manipulation. It is also possible that due to the dogs' temperament, more or fewer signs of pain may be exhibited due to anxiety in the clinic or other factors. Stifle flexion may also not be a very discriminating test in the SFS because many dogs maintained normal stifle flexion regardless of their SI. This is consistent with other studies that have suggested dogs with greater degrees of lameness generally also have decreased extension, but usually have normal stifle flexion (22). It should be noted that while some studies show variable results when measuring thigh circumference, the thigh circumference technique used in this study has demonstrated good repeatability (9).

While the SFS was developed to assess patient function in a clinical environment with the goals of ease of use, obtaining accurate results, and minimal amount and cost of equipment, it does require a goniometer and Gulick II tape measure. This equipment is affordable and easily accessible to veterinarians and physical therapists, and the use of the equipment is feasible in determining the SFS.

We are aware of two other stifle injury scores in veterinary medicine (14, 16, 17). Both testing instruments have been used to compare normal dogs to dogs after surgical correction of CCLR. One of these scores has been used to detect stifle dysfunction and develop a numerical cut-off value between “adequate” and “compromised” stifles (14). Furthermore, this study was not performed with blinding relative to the stifle condition, and this scoring system had the weakest sensitivity and specificity with GRF measures. The other scoring system compared different surgical techniques during the healing process with evaluations 1, 3, and 6 months after surgery (16, 17). Because the scoring system used in this study had owner assessment as a large part of the score and the comparison of dogs 1 month after surgery compared to normal dogs, there is the potential for tremendous bias in scoring these patients. Although we cannot definitively state that this SFS is superior to others, we believe that our SFS is an improvement over these other scores because of comparison to normal dogs, the blinding incorporated in the study design, the evaluation of dogs at random times during recovery, and the comparison to and high correlation with objective weight-bearing using GRFs. Therefore, this SFS may be useful to evaluate a dog's progress throughout injury, recovery, and rehabilitation rather than as a diagnostic tool for cruciate disease or to evaluate different surgical techniques. The use of this score may allow clinical decision-making regarding alterations in activity for a patient. In addition, the proposed SFS uses functional tests and clinical measures and does not include an owner questionnaire as one of the scoring systems does. While the hope is that the SFS may eventually be used to assess dogs with other stifle conditions such as patella luxation, osteochondritis dissecans, and osteoarthritis; we chose the evaluation of unilateral cranial cruciate ligament disease for the initial study to restrict the variable of other stifle conditions. We hope that the SFS can be further tested using other clinical conditions to validate its use for other conditions.

One limitation of this study is the low number of participating dogs as a result of the suspension of elective orthopedic procedures at the hospital during the COVID pandemic. Despite limited patient enrollment, we believe that evaluation of the SFS was sufficiently robust, and confirmed with appropriate statistical tests, to allow recommendations to use the instrument to assess patient disability and perhaps assess patient progress. Another limitation is that dogs were assessed at different stages of recovery from CCLR. This was by design to allow assessment of dogs during different stages of stifle dysfunction. We believe that evaluation of dogs at various stages of cranial cruciate ligament disease, including presurgical and postsurgical cases, strengthens the usefulness of the SFS when comparing the score with objective GRFs, which was the primary objective of this study. Because a heterogenous population was used to look at various stages of stifle injury and recovery, other confounding factors of stifle injury such as meniscal injury or severity of degenerative joint disease were not considered, but it is likely that they contributed to decreased GRFs and SFS values. The purpose of the study was not to evaluate the outcome or chronicity of the disease, but to evaluate if the score was a valid indicator of the degree of lameness and function. Future research may use the SFS to evaluate other factors involved in cruciate ligament disease, including the condition of the meniscus and degree of osteoarthritis, and also recovery from surgery and the evaluation of postoperative rehabilitation programs. Based on our results, the proposed SFS is a relatively sensitive instrument for the clinical evaluation of stifle function and may be able to identify more subtle changes as compared to other scoring systems. Ideally, the SFS would be a useful tool to measure a dog's progress throughout injury and rehabilitation rather than a diagnostic tool to distinguish between normal and abnormal dogs. While some dogs were scored at least two times during the recovery, there were not adequate numbers to make inferences regarding the usefulness of the SFS to monitor progress. But based on the high correlation of the SFS with SI, it is suspected that it would be a useful tool. However, further evaluation of its utility as a clinical tool must undergo additional rigorous testing, including determination of intraobserver and interobserver variability and correlation with GRFs before incorporating this scoring system into global use.

Despite the limitations of the study reported here, a quantitative SFS was developed and effectively tested in dogs with unilateral cranial cruciate ligament disease. Our results support the use of the SFS in a clinical environment to assess disability in dogs following cranial cruciate ligament disease with minimal equipment.

## Data availability statement

The raw data supporting the conclusions of this article will be made available by the authors, without undue reservation.

## Ethics statement

The animal study was reviewed and approved by Institutional Animal Care and Use Committee. Written informed consent was obtained from the owners for the participation of their animals in this study.

## Author contributions

All authors listed have made a substantial, direct, and intellectual contribution to the work and approved it for publication.

## Conflict of interest

The authors declare that the research was conducted in the absence of any commercial or financial relationships that could be construed as a potential conflict of interest.

## Publisher's note

All claims expressed in this article are solely those of the authors and do not necessarily represent those of their affiliated organizations, or those of the publisher, the editors and the reviewers. Any product that may be evaluated in this article, or claim that may be made by its manufacturer, is not guaranteed or endorsed by the publisher.
