# Supplementary material for: Development and testing of a stifle function score in dogs
Source: Front Vet Sci. 2022 Jul 25;9:895567. doi: 10.3389/fvets.2022.895567 (PMC9358000; doi:10.3389/fvets.2022.895567)
Supplement: Supplementary file 1 [file Data_Sheet_1.docx]

Supplementary Appendix 1: Proposed stifle function score

| **Category** | **Section** | **Findings** | **Max Score** | **Score** |
| --- | --- | --- | --- | --- |
| Limb use at a walk | Normal  Mild disuse  Moderate disuse  Severe disuse  Very severe disuse | No lameness and weight bearing on all strides  Lame but weight bearing on >95% of strides  Lame but weight bearing on >50% and <95% of strides  Lame but weight bearing on <50% and >5% of strides  Continuous non-weight-bearing lameness or weight bearing on <5% of strides | 10 | 10  6  4  2  0 |
| Limb use at a trot | Normal  Mild disuse  Moderate disuse  Severe disuse  Very severe disuse | No lameness and weight bearing on all strides  Lame but weight bearing on >95% of strides  Lame but weight bearing on >50% and <95% of strides  Lame but weight bearing on <50% and >5% of strides  Continuous non-weight-bearing lameness or weight bearing on <5% of strides | 10 | 10  6  4  2  0 |
| Lameness at a walk | None  Slight    Mild  Moderate    Severe  Very severe | Normal locomotion  Walks with a slight (barely perceptible) lameness, but strides appear to have normal length  Walks with a mild lameness, but strides appear to have normal length  Walks with a moderate (obvious) lameness or a shortened stride length on affected side when walking, but is bearing weight on that limb  Is intermittently non-weight bearing on that limb when walking  Is completely non-weight bearing on that limb when walking | 10 | 10  8    6  4    2  0 |
| Lameness at a trot | None  Slight    Mild  Moderate    Severe  Very severe | Normal locomotion  Trots with a slight (barely perceptible) lameness, but strides appear to have normal length  Trots with a mild lameness, but strides appear to have normal length  Trots with a moderate (obvious) lameness or a shortened stride length on affected side when trotting, but is bearing weight on that limb  Is intermittently non-weight bearing on that limb when trotting  Is completely non-weight bearing on that limb when trotting |  | 10  8    6  4    2  0 |
| Stance | Normal  Mild asymmetry  Moderate asymmetry  Severe asymmetry | Stands with equal weight on both pelvic limbs  Bears less weight on the affected pelvic limb or limb trembles when standing  Puts limb down for balance but bears weight <10% of normal weight  Does not bear weight on affected limb while standing | 10 | 10  6  4  0 |
| Stair Climbing | Normal  Mild  Moderate  Severe | No difficulty  Has slight difficulty climbing steps  Skips steps or bunny hops  Cannot climb stairs | 5 | 5  3  1  0 |
| Sit-to-stand | Normal    Mild  Moderate  Severe | Easily goes from a sitting to a standing or a standing to sitting position.  Sits and rises squarely  Sits or stands with some difficulty (slight hesitation or delay)  Sits or stands with difficulty (hesitation or delay)  Cannot sit or stand without assistance | 5 | 5    3  1  0 |
| Dancing | Normal  Mild anomaly  Severe anomaly | Moves freely forward and backward  Resists moving forward and backward  Unable to bear weight on pelvic limbs during forward and backward dancing motion | 5 | 5  3  0 |
| Pain response (palpation) | None  Mild  Moderate    Severe | No pain response is elicited during palpation of the joint  Mild pain response (i.e. head turning) is elicited during palpation of the joint  Moderate pain response (i.e. slight vocalization, increased reaction) is elicited during palpation of the joint  Severe pain response (i.e. immediate reaction, loud vocalization, attempt to bite) is elicited during palpation of the joint | 5 | 5  3  1    0 |
| Stifle Effusion | None  Mild  Moderate  Severe | No effusion of stifle  Slight loss of patella ligament distinctness  Patella ligament not distinct  Cannot distinguish patella ligament due to effusion | 5 | 5  3  2  1 |
| Muscle atrophy | None  Mild  Moderate  Severe | Normal muscle mass  Thigh girth is 1% to 5% smaller than the opposite limb  Thigh girth is 6% to 10% smaller than the opposite limb  Thigh girth is >11% smaller than the opposite limb. | 10 | 10  6  4  0 |
| Stifle extension | Normal  Mild loss  Moderate loss  Severe loss | Extension 160° or more  Extension 150°-159°  Extension 140°-149°  Extension <139° | 5 | 5  3  1  0 |
| Stifle flexion | Normal  Mild loss  Moderate loss  Severe loss | Flexion 45° or less  Flexion 46°-50°  Flexion 51°-60°  Flexion >60° | 5 | 5  3  1  0 |
| Cranial Tibial Thrust | None  Mild  Moderate  Severe | Less than 2mm  2-4mm  5-7mm  >7mm | 5 | 5  3  1  0 |
| Total Score |  |  | 100 |  |
